# Effects of work ability and health promoting interventions for women with musculoskeletal symptoms: A 9-month prospective study

**DOI:** 10.1186/1471-2474-9-105

**Published:** 2008-07-21

**Authors:** Agneta Larsson, Lena Karlqvist, Gunvor Gard

**Affiliations:** 1 Department of Health Sciences, Luleå University of Technology, SE-971 87 Luleå, Sweden; 2 Department of Health Sciences, Mid Sweden University, SE-831 25 Östersund, Sweden; 3 Department of Health Sciences, Lund University Hospital, SE-221 85 Lund, Sweden

## Abstract

**Background:**

Women working in the public human service sector in 'overstrained' situations run the risk of musculoskeletal symptoms and long-term sick leave. In order to maintain the level of health and work ability and strengthen the potential resources for health, it is important that employees gain greater control over decisions and actions affecting their health – a process associated with the concept of self-efficacy. The aim of this study was to describe the effects of a self-efficacy intervention and an ergonomic education intervention for women with musculoskeletal symptoms, employed in the public sector.

**Methods:**

The design of the study was a 9-month prospective study describing the effects of two interventions, a comprehensive self-efficacy intervention (*n *= 21) and an ergonomic education intervention (*n *= 21). Data were obtained by a self-report questionnaire on health- and work ability-related factors at baseline, and at ten weeks and nine months follow-up. Within-group differences over time were analysed.

**Results:**

Over the time period studied there were small magnitudes of improvements within each group. Within the self-efficacy intervention group positive effects in perceived work ability were shown. The ergonomic education group showed increased positive beliefs about future work ability and a more frequent use of pain coping strategies.

**Conclusion:**

Both interventions showed positive effects on women with musculoskeletal symptoms, but in different ways. Future research in this area should tailor interventions to participants' motivation and readiness to change.

## Background

Health promotion is an important issue, with the aim of maintaining the level of health and work ability and strengthening the potential resources for health. Health involves a dynamic balance between individuals and their environment, including all individuals' capacity to live and achieve their potential physically, mentally and socially [1-3]. Health promotion in the workplace is a multidimensional concept, where health can be seen as a dynamic balance between employee resources, such as individual capacities, health practices, attitudes and values in relation to psychosocial and organisational workplace factors [1]. Health promoting interventions need to target managers who make qualified decisions regarding structural factors, as well as employees' individual skills, cautiousness and power to influence and act here and now [1,4]. In this article focus is placed on the employees' perspective and on the processes through which individual resources can be strengthened.

According to Antonovsky's salutogenic model, health is seen as a movement along a continuum between ill health and excellent health [5]. Research on health promotion shows the importance of focusing on healthy aspects, for example defining oneself as in good health, having the ability to ignore pain and believing that physical activity does not exacerbate the symptoms [6]. Experiencing trust, team spirit, work pride and confidence [7] are also health- and work ability-promoting factors. For successful return to work, perceived self-efficacy to perform physical tasks, meet role expectations, obtain support and maintain job security is of central importance [8]. Thus, personal resources such as one's ability to assess and understand the situation, to find a meaning in moving in a health promoting direction and having the capacity to do so, seem to function as 'brokers' that moderate how health is affected by stressful situations [5,7]. The demand-control-social support model also indicates that these relations are very important for good health [9,10]. The process through which people gain greater control over decisions and actions affecting their health is frequently associated with Bandura's concept of self-efficacy, i.e. one's confidence in performing a particular behaviour and in overcoming barriers to that behaviour [11,12]. Several studies have been published on the effectiveness of self-efficacy-enhancing interventions on self-management effectiveness and work ability among patients with various chronic diseases [13-15] and it has been identified as important for employees with musculoskeletal pain [1,8,16]. Behavioural interventions focusing on graded activity exposure and skills training [17], on motivating factors such as feedback and rewards, and cognitive processes such as goal formulation, problem solving and information processing [3,18,19] have also been shown to be important for health and work ability. What a person wants is clearly connected with views on one's own possibilities and own competence, what one 'can manage'[20]. Despite the increasing evidence of the importance of employees' self-efficacy for managing musculoskeletal pain and work demands, it has rarely been targeted in workplace-based interventions for employees with musculoskeletal pain [1,8,16]. A 9-month prospective study was designed with the aim of describing the effects of a self-efficacy intervention and an ergonomic education intervention for women with musculoskeletal symptoms, employed in the public sector. The research questions addressed were: 1) what changes in work ability-related factors could be shown within each intervention over the time period, and 2) what changes in health-related factors could be shown within each intervention over the time period?

## Methods

### Study design and subjects

**Figure 1:**
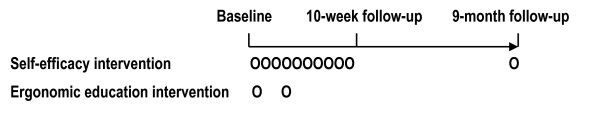
Study design.

**Figure 2:**
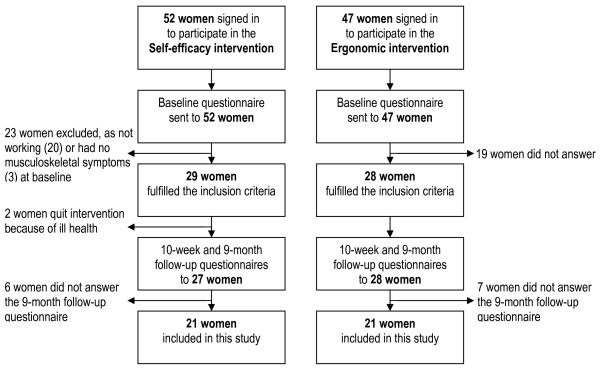
Flow chart of the total number of included and excluded cases, and dropouts during the period studied.

The study was a prospective 9-month follow-up study on the effects of a self-efficacy intervention and an ergonomic education intervention (Figure 1). Approximately 3200 women were employed in public service workplaces within a municipality in the north of Sweden. Invitations to participate in both interventions were sent out to these employees through the first line management in the workplaces. Invitations were also given directly to employees on part-time sick leave by the personnel department. Participation in both interventions was voluntary. The employees selected which intervention they wanted to participate in according to their own interests and motivation and signed on to a list administrated by the personnel department. Both interventions were conducted during paid working hours and the personnel department covered any expenses that arose in terms of cover. The self-efficacy intervention lasted for ten weeks and the ergonomic education intervention for two weeks. Four self-efficacy groups and ten ergonomic education groups were treated during a period of one year. Information of the study was given to all participants in these groups. Thereafter, those who volunteered to take part in the study gave informed consent and answered the baseline questionnaire administered by the University. Inclusion criteria for this study were being female, employed within the public sector, experiencing musculoskeletal symptoms and working at least part-time at the time of baseline measurement. Only those who answered both the baseline questionnaire and the follow-up questionnaires were included (Figure 2). The baseline data are summarised in Table 1 and supplementary baseline values in Tables 2 and 3.

**Table 1:** Baseline data. Individual characteristics, field of work, work attendance, motivation and satisfaction with work and life at baseline for the two groups.

	**Self-efficacy group**	**Ergonomic group**	**p**
Female	*n *= 21	*n *= 21	
Age (years)	46 (33–58)	44 (23–61)	0.308
Height (cm)	167 (154–173)	169 (155–184)	0.632
Body mass index (in kg/m^2^)	24.3 (19.7–40.4)	23,8 (18.3–40.8)	0.675
Musculoskeletal symptoms last 7 days (yes)	*n *= 18	*n *= 20	0.299
Musculoskeletal symptoms related to work (yes)	*n *= 12	*n *= 12	0.675
Seniority (years)	17.5 (2.5–35)	8.5 (1.5–38)	**0.017**
Work field – people	*n *= 14	*n *= 10	
- things	*n *= 5	*n *= 2	
- data	*n *= 2	*n *= 9	
Attendance at work (%)	50 (1–100)	95 (1–100)	**0.021**
- working full time	*n *= 5	*n *= 10	
- working 60 – 95%	*n *= 5	*n *= 9	
- working ≤ 50% ***a***	*n *= 11	*n *= 2	
- no work activity	*n *= 0	*n *= 0	
Sick leave duration (number of months) ***b***	4.2 (0–26)	16 (0–33)	1.000
Satisfaction, in life (1–5)	4 (1–5)	4 (3–5)	**0.011**
Satisfaction, in work (1–5)	3 (3–5)	4 (1–5)	0.502
Motivation, in life (0–10)	7 (2–10)	8.5 (2–10)	0.173
Motivation, in work (0–10)	6 (2–10)	7.5 (2–10)	0.192

Data are given as medians (min-max) or *n *= frequency.

***a ***The frequency of subjects included in ***b ***duration of sick leave

**Table 2:** Changes in work ability-related factors. Within-group changes after ten weeks and nine months compared with baseline.

	**Baseline**	**p 1**	**10-week follow-up**	**9-month follow-up**	**p 2**	**p 3**
**Work ability index (WAI) **(7–49)						
Self-efficacy group Ergonomic group	28 (17–47) 38 (16–49)	**0.023**	31 (20–49) 39.5 (16–49)	34 (20–48) 40.5 (17–49)	0.574 0.896	**0.028** 0.983
1. **WA relative to lifetime best **(0–10)						
Self-efficacy group Ergonomic group	5 (0–10) 7 (0–10)	0.063	6 (1–10) 8 (0–10)	7 (0–10) 8 (0–10)	0.290 1.000	0.129 0.833
2a. **WA/physical demands **(1–5)						
Self-efficacy group Ergonomic group	3 (1–5) 4 (2–5)	**0.008**	3 (2–5) 4 (1–5)	3 (2–5) 4 (1–5)	**0.021** 0.405	**0.012** 0.265
2b.**WA/mental demands **(1–5)						
Self-efficacy group Ergonomic group	3 (2–5) 4 (1–5)	**0.016**	3 (2–5) 4 (1–5)	3 (2–5) 4 (2–5)	0.206 1.000	0.052 0.808
3.**Diagnosed diseases **(1–7)						
Self-efficacy group Ergonomic group	5 (2–7) 5 (4–7)	0.501	5 (2–7) 5 (3–7)	5 (3–7) 6 (3–7)	0.458 0.366	0.408 0.458
4. **Work impairment **(1–6)						
Self-efficacy group Ergonomic group	2 (1–6) 4.5 (1–6)	**0.042**	4 (1–6) 5 (1–6)	4 (1–6) 4.5 (1–6)	**0.047** 0.120	0.119 0.809
5. **Sickness absence **(1–5)						
Self-efficacy group Ergonomic group	2 (1–5) 4.5 (1–5)	**0.000**	2 (1–5) 4 (1–5)	3 (1–5) 4 (1–5)	0.705 0.248	0.058 0.250
6. **Belief of work ability **(1,4,7)						
Self-efficacy group Ergonomic group	7 (1–7) 7 (1–7)	0.858	4 (1–7) 4 (1–7)	7 (1–7) 7 (1–7)	0.102 0.564	0.655 **0.046**
7. **Psychological well-being **(1–4)						
Self-efficacy group Ergonomic group	3 (2–4) 3 (1–4)	0.573	3 (1–4) 3 (1–4)	3 (2–4) 3 (1–4)	0.782 0.739	0.052 0.100
						
**Physical strain in work **(6–20) *						
Self-efficacy group Ergonomic group	14 (9–18) 12 (6–15)	**0.003**	14 (9–17) 12 (6–16)	15 (7–17) 12 (6–16)	0.130 0.459	0.279 **0.044**
						
**Coping in relation to work**						
1. **Problem-focused coping **(0–100)						
Self-efficacy group Ergonomic group	75 (50–100) 75 (50–100)	0.596	75 (50–100) 75 (50–100)	88 (50–100) 75 (38–100)	0.672 0.236	0.351 0.714
2. **Selective coping **(0–100)						
Self-efficacy group Ergonomic group	38 (0–100) 38 (0–75)	0.556	38 (0–88) 44 (0–62)	38 (0–75) 50 (12–75)	0.420 0.484	0.174 0.109
3. **Resigning coping **(0–100) *						
Self-efficacy group Ergonomic group	25 (0–88) 38 (0–62)	0.828	25 (0–88) 38 (0–75)	25 (0–50) 38 (0–62)	0,138 0.541	0.060 0.542

* High values represent **a bad level **for the items 'physical strain in work' and 'resigning coping'.

**A high value **is **good **for all other items.

Data are given as medians (min-max) and as differences within groups.

p 1 = differences between the groups at baseline (Mann Whitney U)

p 2 = baseline compared with 10-week follow-up within the groups (Wilcoxon Signed Ranks Test)

p 3 = baseline compared with 9 month follow-up within the groups (Wilcoxon Signed Ranks Test)

**Table 3:** Changes in health-related factors. Within-group changes after ten weeks and nine months compared with baseline.

	**Baseline**	**p 1**	**10-week follow-up**	**9-month follow-up**	**p 2**	**p 3**
**General health**						
1: **Severity of symptoms **(0–10) *						
Self-efficacy group Ergonomic group	6 (3–9) 5 (0–8)	0.083	5 (3–7) 4 (1–10)	5 (1–10) 5 (2–10)	**0.023** 0.819	0.113 0.924
2: **State of health **(1–5)						
Self-efficacy group Ergonomic group	3 (2–4) 4 (2–5)	0.224	3 (2–5) 4 (1–5)	4 (2–5) 4 (2–5)	0.265 0.793	0.097 0.782
3: **Mental strain **(1–5) *						
Self-efficacy group Ergonomic group	3 (1–5) 3 (1–5)	0.455	3 (1–4) 3 (1–5)	2 (1–4) 2 (1–4)	0.776 0.297	0.070 0.169
						
**Coping in relation to pain**						
1: **Positive distraction **(0–6)						
Self-efficacy group Ergonomic group	3.5 (1.5–5.5) 2.5 (0–4.5)	0.082	3 (0–5) 3 (1.5–5)	3 (0–6) 3.5 (2.5–4.5)	0.681 0.059	0.134 **0.002**
2: **Catastrophic thinking **(0–6) *						
Self-efficacy group Ergonomic group	3 (1–6) 2 (0–4.5)	0.051	2.5 (1–6) 2.5 (0–4.5)	3 (0.5–4.5) 2 (0–4.5)	0.482 0.352	0.237 0.784
3: **Ignoring pain **(0–6)						
Self-efficacy group Ergonomic group	3 (0–5) 3.5 (1–5)	0.087	3.5 (1.5–6) 3.5 (1.5–5.5)	3.5 (1–4.5) 4.2 (1.5–5.5)	0.052 0.310	1.000 **0.048**
						
**Self-efficacy in relation to pain**						
1: **Control pain **(0–6)						
Self-efficacy group Ergonomic group	4 (0–5) 3 (2–6)	0.947	4 (2–5) 4 (3–6)	3.5 (0–5) 4 (2–6)	0.577 **0.040**	0.366 0.071
2: **Reduce pain **(0–6)						
Self-efficacy group Ergonomic group	3 (1–5) 3 (2–6)	0.422	4 (2–6) 4 (3–5)	3 (1–6) 4 (2–6)	0.054 0.130	0.913 0.580

* High values represent **a bad level **for the items 'severity of symptoms', 'mental strain' and 'catastrophic thinking'.

**A high value **is **good **for all other items.

Abbreviations as in Table 2.

At baseline, no significant differences were found between participants in the two interventions in terms of age, body height, BMI, presence of musculoskeletal symptoms and their relation to work, satisfaction with present work, motivation for change in work or private life. However, some differences were connected with the participants' opportunity to select which intervention they wanted to participate in. Participants in the self-efficacy intervention were less satisfied with their present life situation, had higher seniority and lower attendance at work; eleven worked reduced hours because of sick leave and five had part-time jobs. The majority worked with *people*, for example as a teacher, child minder or assistant nurse while about a quarter worked with *things*, for example as a cleaner or cook. In the ergonomic education intervention two women worked less due to sick leave and nine had part-time jobs. Half of the participants worked with *people *and the other half with *data*, for example clerical and customer service jobs (Table 1).

Baseline values in work ability-related factors (Table 2) showed no significant differences between the two groups in terms of use of coping strategies at work, psychological well-being or in positive belief about future work ability. The self-efficacy group showed a significantly lower self-reported work ability (total WAI score) than the ergonomic group. The self-efficacy group also perceived a significantly lower work ability in relation to physical and mental demands, higher work impairment and sickness absence and higher physical strain at work. Baseline values in health-related factors (Table 3) showed no significant differences between the two groups in terms of perceived state of health, in mental strain or in any other factor.

### Interventions

#### A. Self-efficacy intervention

The aim of the self-efficacy intervention was to promote health and work ability by improving individual self-efficacy, priority-making, self-reflection, empowerment, coping skills, physical activity patterns and insight into one's own life situation. The intervention consisted of ten weekly group sessions as well as physical activity, followed by individual practice in the life and work situation for an additional six months, with a follow-up session at the end of this time. Each group session lasting three hours was conducted by a psychologist with groups of about ten participants. These sessions consisted of group discussions and self-reflections in relation to different topics and to the participants' own life situations, i.e. 'what does this mean for me?' The discussions were combined with education by invited specialists in different topics: physical activity, diet, psychological stress and strain, mental training, working environment factors, insurance factors and social insurance office liability. As a parallel part of the intervention each participant practised physical activity 2–3 hours a week. These activities were individually tailored to physical capacity and were supported by physiotherapists and mentors, and during the first three months free training sessions at a training centre were offered.

#### B. Ergonomic education intervention

The aim of the ergonomic education was to promote health and work ability by improving self-management skills, coping with pain at work, ergonomic and preventive knowledge about work environment factors and how to perform necessary changes. The intervention was conducted by a physical therapist in the occupational health service, in groups of about four participants with similar musculoskeletal problems. The group met at two monthly three-hour sessions and received education about ergonomic and psychosocial work issues in relation to work and health and the practice of stretch-and-flex breaks, physical activity and relaxation.

### Questionnaire

Data were obtained through a self-report questionnaire with 43 questions from reliable and valid questionnaires and one question concerning attendance at work, developed by the authors. Baseline data were assessed with questions about gender, age, body height, weight, period of time working in current job and seniority, profession, principal work tasks and field of work [21-23]. This provided a basis for classification into different work categories (people/things/data) [24,25]. Five-point Likert scales were used to estimate current state of health (1 = 'very bad', 5 = 'very good') [23], mental strain and satisfaction with current work and life situation (1 = 'not at all/very bad/very unpleased', 5 = 'a lot/very good/very pleased') [22,26]. The presence of musculoskeletal symptoms during the previous seven days and their relation to the present work situation were assessed with two questions (response options yes/no) [21]. Eleven-point visual analogue scales (VAS) were used for rating the intensity of current musculoskeletal symptoms (0 = 'no symptoms' to 10 = 'worse imaginable symptoms') [27] and current motivation to set about necessary changes in their working and living conditions (0 = 'very bad' to 10 = 'very good') [28].

Work ability was assessed by ten questions forming seven items of the Work Ability Index (WAI) [29,30] (alpha = 0.87): 1) current work ability compared with lifetime best (0 = 'poor' to 10 = 'excellent'); 2) work ability in relation to the physical and mental demands of the work (2 = 'very bad' to 10 = 'very good'); 3) number of current diagnosed diseases (1 = '≥ five' to 7 = 'none'); 4) estimated work impairment due to diseases or illnesses (1 = 'total' to 6 = 'none'; 5) sickness absence during the past 12 months (1 = '>100 days' to 5 = 'none'); 6) belief about work ability in present occupation two years from now (1 = 'no', 4 = 'maybe' or 7 = 'yes'); and 7) psychological well-being (1 = 'never' to 4 ='often'). The WAI score ranged from 7 to 49 points, with a score at, or below, 36 points indicating low work ability. In the present study item 3 included a smaller number of illnesses than the original WAI, and at the end the question 'state if you have any disease, illness or handicap' was included, as suggested previously [31]. To discriminate physical and mental demands the two questions forming item 2 are presented separately. The participants' ordinary physical strain at work was graded on a Borg RPE-scale (range 6–20) [23].

Coping strategies in working life, e.g. 'what do you usually do when problems arise at work?' were assessed on three scales taken from the Copenhagen Psychosocial Questionnaire [32]: *problem-focused *coping: 'do you try to find out what you can do to solve the problem?'(alpha = 0.75); *selective *coping: 'do you try to think of something else or do something you like?'(alpha = 0.62); and *resigning *coping: 'do you accept the situation because there is nothing you can do about it anyway?'(alpha = 0.63). Each item comprised five responses (0 = 'never', 100 = 'always').

Coping abilities for pain were assessed by a single item from each of the eight subscales in the Coping Strategies Questionnaire (CSQ) [33,34]. As in previous item-level studies [35,36] factor analysis of the single items revealed three subscales that were factorially distinct and internally consistent: 1)*Positive distraction *comprised the two items 'I think of things I enjoy doing' (diverting attention) and 'I leave the house and do something active' (increased behavioural activities) (alpha = 0.55). 2) *Catastrophic thinking *comprised the two items 'It's awful and I feel that it overwhelms me' (catastrophising) and 'I take my medication' (pain behaviours) (alpha = 0.67). 3) *Ignoring pain *comprised the two items 'I tell myself I can't let the pain stand in the way of what I have to do' (coping self statements) and 'I ignore it' (ignoring sensations) (alpha = 0.66). Each item was graded on a seven-point Likert scale (0 = 'never do when in pain', 6 = 'very frequently do when in pain') and the items within each factor were summed and averaged to form the scales. Two single items did not load consistently on any factor and were excluded. Self-efficacy was consistent with the original CSQ, rated by two items on seven-point Likert scales: 1) 'control over pain' and 2) 'ability to decrease pain'.

### Statistical analysis

Median (Md), min-max values and prevalence (%) were used for descriptive statistics. The Mann-Whitney *U *test was used for between-group comparisons at baseline and the Wilcoxon signed-rank test was used for within-group changes after ten weeks and nine months compared with baseline. Due to small sample sizes p-values ≤ 0.05 were considered statistically significant. Principal component factor analyses and analyses of internal reliability were used to test each scale in relation to our study population, and the values are presented in the Methods section. Chronbach's alpha values above 0.6 were considered to indicate a sufficient degree of internal consistency for the scales [37]. The statistical analyses were performed using the Statistical Package for the Social Sciences (SPSS) software version 11.5.

### Ethics

The study was regarded as quality development work within the area of occupational health. The aims, methods, and procedures of the study were developed in cooperation with and agreed by the occupational health service and the personnel department of the municipality. The study was performed in compliance with the ethical principles of the Helsinki Declaration. Decline participation in the study or to withdraw consent to participate did not affect the employees opportunity to take part in the interventions.

## Results

### Changes in work ability-related factors within each intervention over the time period

#### Results within the self-efficacy intervention group

At baseline 16 of the 21 subjects were classified as having low work ability compared with 12 subjects at the 9-month follow-up, indicating a statistically significant improvement. The sub score 'work ability in relation to physical demands' was also significantly improved. Ten subjects stated that they had a *fairly good *(score 3) balance at baseline and at follow-up. The number of subjects stating a *pretty *or *very good *balance (score 4 and 5) increased from five at baseline to nine at nine months. After ten weeks the work ability in relation to physical demands had increased. At ten weeks significant improvement was also noted in terms of less work impairment due to disease or illness. No other changes were noted (Table 2).

#### Results within the ergonomic education intervention group

At nine months there was no change in the total WAI score. At baseline 13 of the 21 subjects had positive beliefs (score 7) in their ability to work in their present occupation two years from now, and at nine months 17 subjects had this belief, which was a statistically significant improvement. Physical strain at work was also significantly increased. No changes were noted at ten weeks (Table 2).

### Changes in health-related factors within each intervention over the time period

#### Results within the self-efficacy intervention group

No significant changes were noticed at nine months. At ten weeks the intensity of musculoskeletal symptoms was significantly reduced (Table 3).

#### Results within the ergonomic education intervention group

Significantly more frequent use of the pain coping strategies 'positive distraction' and 'ignoring pain' was found at nine months. The median values rose to 3.5 from 2.5 and to 4.2 from 3.5 respectively. The use of catastrophic thinking was unchanged, remaining at a median value of 2. At ten weeks a significant increase in self-efficacy to control pain was noted (Table 3).

## Discussion

Over the time period studied there were small magnitudes of improvements within each group. The small improvements that appeared differed between the two groups. The major effect in the self-efficacy intervention group was increased perceived work ability, reflected in the total WAI score and its sub score 'work ability in relation to physical demands'. The ergonomic education intervention showed more frequent use of pain coping strategies, and an increased number of subjects with positive beliefs in their ability to work in their present occupation.

These differences in outcomes may to some extent reflect the fact that the interventions attracted participants' with somewhat different starting points, as shown in the baseline values. The participants' opportunity to select which group they wanted to participate in implied motivated participants in both interventions. This condition was considered a prerequisite for fulfilling the aim of the interventions. In the present study 16 women (76%) in the self-efficacy group and nine women (43%) in the ergonomic group had low work ability at baseline (at, or below, 36 points) according to the WAI. This is high compared with the frequency (25%) of low work ability in the female working population [23]. It has been proposed that the WAI can identify subjects with low work ability who are in need of more extensive support, while ergonomic education is recommended for subjects with higher scores [29]. This is only partly in accordance with the participants' own choice of intervention in this study. A major focus, especially in the self-efficacy intervention, was the motivation to work with oneself and one's life situation. Besides lower work ability, those who wanted to participate in the self-efficacy intervention were, to a higher extent than the ergonomic group, employed within physically strenuous *people *or *thing *occupations and were experiencing less life satisfaction. It may be that these participants chose to attend the comprehensive self-efficacy intervention, as they needed more social support, coaching and help to make work and life-style changes. They may have needed to participate in a process of reflection and awareness to make priorities in their own life situation. This reasoning is supported by studies showing that persons on sick-leave may experience reduced self-esteem, sense of control, self-determination and feel shame [38,39]. The self-monitoring that takes place in rehabilitation groups, such as in the present study, have positive effects on these issues [38,39]. The encountering process in itself has been shown to be very important. Being met with recognition enhances strength, confidence and awareness which can provide tools with which to handle pain and illness and, as such, is important in the recovery process [38,40]. To listen to other participants describing successful solutions to specific work or life situations may, according to the theories of self-efficacy [11], increase the participants' self-efficacy. An increased perception of own ability to manage work or perform a specific behavioural change can increase work motivation [3,20]. For participants in the present study, the acts of signing on to a list for intervention was one step in a health promoting direction. It has previously been shown among employees that factors such as feeling susceptible to work-related musculoskeletal disorders and experiencing pain influences their will to take actions to improve their health and work situation. Working on one's readiness to change may influence the receptiveness of health information and education and promote positive behaviour changes [41], as in this study.

### Changes in work ability-related factors

The self-efficacy group showed positive effects on work ability that were reflected in the WAI, with the sub score 'work ability in relation to physical demands' improving the most. These changes could be explained by increased physical capacity in relation to work demands and/or an increased power and ability to control participants' own life situations. Physical activities were a part of both our programmes, but the self-efficacy group had, to a greater extent, physical activities individually tailored to their physical capacities and were supported by physiotherapists and mentors. Previous research has shown the positive effects of physical activity interventions on musculoskeletal symptoms and sick leave [42,43]. Pain-related beliefs such as high self-efficacy and low fear-avoidance have been shown to be important determinants of work ability among patients with musculoskeletal pain. The intensity of pain has produced conflicting results, but has been found to have a more negative impact on work ability in women and among those listed as sick [16,44]. Interventions that focus on participants' self-confidence and self-efficacy in dealing with symptoms and work-related problems have proved to be effective [13-15]. Finally, the higher the level of control and ability to influence one's situation, the greater the opportunity for health and work ability [9,10].

The ergonomic education group showed increases in positive beliefs about their ability to work in their present occupation two years from now. Other studies have shown that one's view of one's own competence and expectations of recovery and work ability are important predictors for better health outcomes and work ability [20,45-47]. The ergonomic education group that had a high work ability at baseline may have increased their knowledge and practical ability to solve ergonomic problems at work and increased their self-management of pain in work situations. This is in line with the recommendation for ergonomic education of subjects with higher WAI scores [29]. It has been argued that the benefits of educational programmes may depend on providing social support and encouraging employees' ability and responsibility to solve their own problems [15,17], which, within the scope of the limited number of sessions, was also the intention of the ergonomic education in the present study.

### Changes in health-related factors

No significant improvements were found within the self-efficacy intervention group, except for reduced musculoskeletal symptoms at ten weeks. At this point, descriptive statistics also indicated an increased use of the 'ignoring pain' strategy as well as increased self-efficacy to reduce pain. These effects were not seen at nine months. Other studies have reported declining effects at follow-up due to lack of group support [40]. This points to the importance of different sources of support in life and in work and of a positive working environment in order to attain health and sustained or improved work ability. Supervisors and co-workers attitudes, beliefs and basic knowledge about how to be supportive and how to promote a good working environment is important [48,49]. However, it has been shown that a high proportion of employees, even though they may experience musculoskeletal pain, may not yet realise their need for preventive efforts to reduce work environmental risks and improve their health [41]. Key factors in promoting health and work ability in the work place can involve learning a constructive coping pattern, creating an open work climate, communication and learning [50].

A significantly increased use of active coping strategies was shown at nine months within the ergonomic education group. However, catastrophic thinking was unchanged. At ten weeks the increase in self-efficacy to control pain was significant. The fact that this had no effect on musculoskeletal symptoms or state of health is however puzzling. It has been suggested that treatment programmes that are designed to encourage active coping strategies may encourage passive coping as well [51]. It may also be that different styles of coping are important at different stages of recovery and at different levels of pain severity [51,52]. It has previously been shown that a patient's use of active strategies such as positive distraction and ignoring pain, and their belief in control of pain, are positively associated with general activity level in patients with lower pain levels [52]. Decreased perceived control over pain, belief in oneself as disabled by pain, catastrophising cognitions and increased use of passive strategies, i.e. a tendency to withdrawal or to rely on an outside source, have been shown to be strong negative predictors of daily functioning and should be controlled by cognitive behavioural methods [35,51-53].

Previous research has shown that a cognitive behavioural intervention in itself or in combination with preventive physiotherapy is effective in increasing self-efficacy to control and reduce pain and promote work ability in the long term [54,55]. The timing of an intervention is important, with early return-to-work programmes being more cost-effective than rehabilitation at a later stage [55]. In the present study, even though all participants were working, some of them had been listed as sick part-time for up to 2.5 years, and may have needed this intervention earlier for greater health and work ability effects. This study showed that these two interventions had positive effects on women with musculoskeletal symptoms but in different ways. In spite of the small magnitudes of improvements, these can indicate the beginning of positive development in use of effective coping strategies and in more positive beliefs about recovery and ability to work.

### Limitations of the study

It was not possible to use randomisation or pair-wise matching in the present study. An advantage was that both interventions were selected according to the participants' own interests, need for change and motivation. This selection opportunity produced some differences between the groups at baseline, where the ergonomic education group were somewhat advantaged. An increased number of subjects was desired but this was not possible for organisational and economic reasons. The inclusion criteria 'having musculoskeletal symptoms' and 'working' restricted the sample sizes, but were considered important for this study. The sample size was also limited by subjects not responding to the nine-month follow-up questionnaire. Those that dropped out in general had a longer period of part-time sick leave and reported poorer levels of health-related factors than subjects included in the study. The reason they did not respond to follow-up is unknown. It is possible that the part-time sick leave participants' choice of intervention was influenced by someone in the rehabilitation network (the first line management, social insurance officer, personnel manager, occupational health service), but we have no evidence of this. As the interventions were carried out alongside normal life and work, other factors in the participants' lives may have affected their health and work ability. Since baseline values for some items were relatively high, a ceiling effect may have caused positive changes to be underestimated. To answer our research questions we had to rely on questions from many different standardised questionnaires. Most of the questions used had been tested for reliability and validity and, in addition, a few scales were reliability-tested for use in the present study group. Generalisation of the results is limited to women experiencing musculoskeletal symptoms, and working in mostly female-dominated work settings in the public human service sector.

## Conclusion

Both interventions showed positive effects on women with musculoskeletal symptoms, but in different ways. In general there were small magnitudes of improvements within each group. Positive effects in perceived work ability were found in the self-efficacy intervention group. The ergonomic education group showed effects on increased positive beliefs about future work ability and a more frequent use of pain coping strategies. Future research in this area should tailor interventions to participants' motivation and readiness to change.

## Competing interests

The authors declare that they have no competing interests.

## Authors' contributions

All authors have been involved in the development of the study design. AL was responsible for the data collection and for writing the manuscript. GG and LK participated in the general coordination of the study and read and corrected draft versions of the manuscript.

## Pre-publication history

The pre-publication history for this paper can be accessed here:


